# The clinical features and ^18^F-FDG-PET analysis of absence status epilepsy

**DOI:** 10.3389/fneur.2025.1521842

**Published:** 2025-05-02

**Authors:** Jing-Wen Zuo, Xiao-Qiu Shao, Qun Wang, Rui-Juan Lv

**Affiliations:** Department of Neurology, Beijing Tiantan Hospital, Capital Medical University, China National Clinical Research Center for Neurological Diseases, Beijing, China

**Keywords:** absence status epilepsy, FDG-PET, posterior regions, clinical features, metabolism

## Abstract

Objective To summarize the clinical features and ^18^F-fluorodeoxy-glucose positron emission tomography (^18^F-FDG-PET) patterns of absence status epilepsy (ASE). Methods In our study, three patients with ASE were presented, and a comprehensive review of the relevant literature was conducted to elucidate the clinical features and PET results of patients with ASE. Results Seventeen cases of ASE from 7 articles were identified. A total of 20 cases were included in this study, including 9 males (9/20) and 11 females (11/20). The average age at onset was 28.1 ± 15.07 years. Patients with ASE typically present with prolonged episodes of confusion and unresponsiveness. Some patients also present with generalized tonic clonic seizure (GTCS). The episodic frequency was relatively low, ranging from once per year to once per month, and the duration of each episode varied from 30 min to 3 weeks. The episodes of five patients coincided with menstruation, and one patient experienced episodes triggered by sleep deprivation and emotional disturbance. Ictal electroencephalogram (EEG) revealed generalized spike–wave (SW) activity at 2–4 Hz, and brain magnetic resonance imaging (MRI) revealed normal findings. Two patients underwent ictal ^18^F-FDG-PET, which revealed hypermetabolism in the bilateral thalamus and cerebellar vermis, along with hypometabolism in the bilateral frontal and parietal cortices and cerebellar hemispheres. The three patients in this study underwent interictal ^18^F-FDG-PET, which revealed decreased metabolic activity in the temporal, parietal, and occipital cortices and cerebellum. Furthermore, the patients’ thalamic area and standard uptake value (SUVavg) were lower than those of healthy individuals. Seventeen cases (17/20) became seizure-free after treatment with valproate (VPA) and lamotrigine (LTG). Interpretation ASE can be considered a specific syndrome of genetic generalized epilepsies (GGEs). Interictal brain PET imaging may reveal relative hypometabolism in posterior regions, along with decreased thalamic area and metabolic activity, potentially indicating the key role of posterior regions in sustaining wakefulness. Most patients responded well to VPA and LTG.

## 1 Introduction

Absence status epilepticus (AS) is characterized by prolonged and generalized absence seizures lasting for several hours or even days (1). AS can be described as “typical,” occurring in the context of idiopathic generalized epilepsy (IGE), and “atypical,” occurring in symptomatic or cryptogenic generalized epilepsy (2). In 2008, Genton et al. (3) reported a series of patients with AS as the predominant clinical feature and proposed the term and diagnostic criteria for “absence status epilepsy (ASE).”

The 2017 International League Against Epilepsy (ILAE) classification described the term “genetic generalized epilepsies (GGEs)” as a wide range of epilepsies with generalized seizure types and generalized spike–wave (SW) (4). In addition to IGE, GGEs also include patients with generalized seizure types that do not meet the criteria for a specific syndrome and less commonly generalized epilepsy syndromes (5). Therefore, ASE can be classified as a rare type of GGEs.

ASE may occur in puberty or early adulthood. Patients with ASE present predominantly with recurrent and unprovoked AS, with episodes lasting from 30 min to 3 weeks (3). The most common manifestations include clouding of consciousness, with or without generalized tonic–clonic seizure (GTCS), and unresponsiveness. The ictal electroencephalogram (EEG) in patients with ASE shows 2–4 Hz continuous generalized SW or poly spike–wave (PSW) discharge. Given the infrequent occurrence of ASE and the resemblance of ASE to certain subtypes of GGE, such as *de novo* late onset ASE (dnASLO) and a syndrome of IGE with phantom absences (IGE-PA) (6), there is a risk of patients being misdiagnosed with complex partial seizures or other syndromes, which may lead to inappropriate or ineffective drug treatments. Therefore, earlier identification and definitive diagnosis of ASE are crucial.

^18^F-fluorodeoxy-glucose positron emission tomography (^18^F-FDG-PET) is considered a valuable imaging method for the preoperative evaluation of seizures and the diagnosis of autoimmune encephalitis. Additionally, there are articles available that explore the PET characteristics of patients with ASE (7, 8), offering insights into the underlying mechanisms and PET features of this condition. In this study, we presented three patients with ASE, meeting the diagnostic criteria established by Genton et al. (3). Furthermore, we conducted ^18^F-FDG-PET scans to identify the metabolic features of ASE in these three patients. Additionally, we reviewed existing clinical data from the relevant literature to further investigate the clinical features of ASE.

## 2 Materials and methods

### 2.1 Patient inclusion

Three patients with ASE were retrospectively screened at the Department of Neurology in the Beijing Tiantan Hospital affiliated with Capital Medical University between January 2022 and September 2023. All patients provided written informed consent to participate in the study and for publication of their clinical details. The diagnostic criteria for ASE were consistent with those proposed by Genton et al. (3) and Koutroumanidis et al. (9) and are as follows: (1) recurrent, unprovoked episodes of typical AS representing the unique or predominant seizure type, (2) at least one episode of AS recorded by video-EEG or by EEG only, and (3) clinical and EEG features fulfilling the criteria of IGE (10). According to the International League Against Epilepsy (ILAE) Classification of Epilepsies (4), the diagnostic criteria of IGEs are as follows: (1) IGEs include childhood absence epilepsy (CAE), juvenile absence epilepsy (JAE), juvenile myoclonic epilepsy (JME), and epilepsy with generalized tonic–clonic seizures alone (GTCA). Diagnosis of these seizure types requires EEG documentation. (2) EEG findings typically reveal generalized SW discharges at 2.5–5.5 Hz on EEG. These abnormalities are more obvious during sleepy status or wakefulness, and may be provoked by hyperventilation or photic stimulation. (3) Developmental and cognitive abilities are generally unimpaired. Patients may exhibit comorbidities such as attention deficit hyperactivity disorder (ADHD), learning difficulties, or mood disorders. (4) IGEs typically exhibit a polygenic inheritance pattern, with a familial predisposition, though a clear family history may not be present in all cases. (5) IGEs generally respond well to pharmacological treatment, especially to broad-spectrum antiepileptic drugs (ASMs). However, certain ASMs, such as sodium channel blockers, may exacerbate certain symptoms, including absence or myoclonic seizures. (6) Excluding other types of epilepsy or abnormal discharges, such as focal seizures, potential epileptogenic changes on brain imaging, and suspected paroxysmal focus or more than minor intermittent focal slowing on EEG.

EEG interpretation was evaluated by two neurologists to increase the accuracy of the results. Patients were included by experienced neurologists on the basis of these established diagnostic criteria.

### 2.2 ^18^F-FDG-PET acquisition

Two patients and 10 healthy controls underwent PET/ magnetic resonance imaging (MRI) scans. One patient underwent an interictal PET/computed tomography (CT) scan. PET/CT images were acquired with a PET/CT system (Elite Discovery, GE HealthCare). PET/MR images were acquired with a 3.0 T time-of-flight (TOF) signal PET/MRI (GE Healthcare, Milwaukee, WI, United States). The mechanism underlying the occurrence of absence seizures is thought to be related to the thalamus (11, 12); therefore, we chose to assess the area and metabolic activity of this region. The thalamic area and metabolic value were acquired via MedEx software. First, the PET/MR dataset was loaded, and a tool such as Volume Viewer or PET VCAR was opened. Patient data (weight, injected dose, and scan timing) were entered into the DICOM metadata. Second, ROI tools were used to define the region of interest (e.g., spherical, elliptical, or freehand), and the software calculated SUV metrics such as the SUVmax, SUVmean, and SUVpeak. Third, ROI placement was verified via axial, coronal, and sagittal views, and the results were saved for reporting.

### 2.3 Literature review

We performed a comprehensive and systematic search of the available literature on ASE. The literature retrieval platforms included WanFang Data, CNKI, PubMed, and Web of Science for articles up to December 2023 by using the title/abstract keywords “absence status epilepticus” or “absence status epilepsy” or “ASE” or “non-convulsive status epilepticus.” We selected original studies and case reports that included confirmed patients with ASE, and excluded possible cases or cases of AS not fulfilling the criteria for ASE. Reference screening was conducted by two experienced neurological doctors.

## 3 Results

### 3.1 Case report

#### 3.1.1 Patient 1

**Figure 1:**
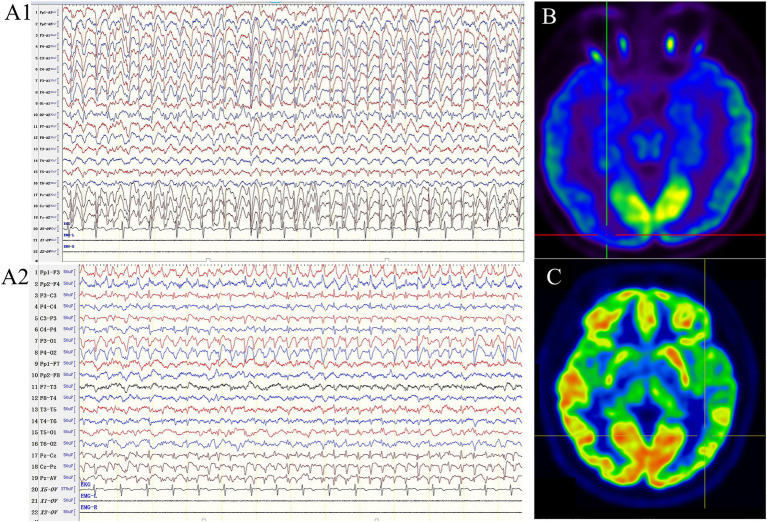
The ictal EEG and PET/MR of our patients. **(A)** The ictal EEG of patient 1, which showed continuous generalized spike–waves at 2.5–3 Hz (A1: ipsilateral ear montage; A2: anteroposterior bipolar montage; sensitivity 10 μV/mm, low frequency filter 1.6 Hz, high frequency filter 70 Hz). **(B)** The PET/MR of patient 1 showed hypometabolism in the right temporal, parietal, occipital lobe, and cerebellum. **(C)** The PET/MR of patient 2 revealed relative hypometabolism in the left temporal, parietal and occipital lobe.

A 38-year-old right-handed female was admitted to the hospital, complaining of recurrent episodes of loss of consciousness and limb twitching for 20 years. She experienced her first GTCS at the age of 18, followed by additional episodes at the age of 21. These episodes were characterized by altered consciousness and dialeptic seizures lasting from several hours to days, she also reported occasionally asymmetric limb twitching with alternating lateralization between the left and right extremities. These episodes recurred every 4–6 months, coinciding mostly with her menstrual period. No abnormalities were observed during the postnatal or developmental stages. Assessments including family history, routine laboratory tests, genetic testing and a 3.0 T MRI were also unremarkable. During the interictal period, she underwent brain PET/MRI, revealing hypometabolism in the right temporal, parietal, and occipital lobes and the cerebellum (shown in Figure 1B). Three days after admission (during her menstrual period), she experienced a prolonged episode lasting approximately 22 h, characterized by a dialeptic state, confusion, and motionlessness. Although the patient subjectively reported occasional leg twitching during episodes, the ictal video electroencephalogram (VEEG) monitoring revealed no evidence of limb twitching during the recorded seizure event. And ictal EEG showed continuous generalized SW or PSW discharge at 2.5–3 Hz (shown in Figure 1A). Seizure activity resolved after intravenous infusion of 10 mg of midazolam (MDZ). The interictal EEG revealed no significant abnormalities.

She started taking valproate (VPA) at the age of 18 and became seizure-free for two and a half years. At age 21, she experienced ASE once every 4–6 months. Thereafter, her medication was adjusted to VPA and oxcarbazepine (OXC), which reduced the frequency of seizures to once a year. At age 35, the seizure frequency increased to once a month, OXC was discontinued and replaced with lamotrigine (LTG), and there was no recurrence of seizures during the 1-year follow-up period.

#### 3.1.2 Patient 2

A 36-year-old female was admitted to our hospital for acute onset of clouding of consciousness and unresponsiveness, which persisted for 18 h. The neurological examination revealed dilated pupils and absent pupillary reaction to light. An urgent VEEG was performed, which demonstrated generalized 2.5–3 Hz SW activity. Subsequently, seizure activity was successfully resolved with intravenous infusion of MDZ.

She had been experiencing ASE since the age of 22, with a progressive increase in frequency occurring approximately once a month within the past year. Almost every episode coincided with her menstrual period. She had no history of other types of epilepsy and had not taken any antiepileptic drugs (AEDs) prior to admission. PET/MRI was conducted after she recovered completely, revealing relative hypometabolism in the left temporal, parietal and occipital lobes (shown in Figure 1C). She has been seizure-free on levetiracetam (LEV) for 3 months.

#### 3.1.3 Patient 3

A 27-year-old right-handed female with a history of seizures since the age of 13 years was admitted to our hospital. She experienced her first GTCS at the age of 13. Eight years ago, she experienced episodes of intermittent limb weakness without clear triggers, manifesting as difficulty in grasping objects and walking, accompanied by unresponsiveness and urinary incontinence. These episodes typically lasted 1–2 days and occurred once or twice per year. Additionally, her seizures occurred during her menstrual period.

The family history, laboratory test results, and brain MRI findings were unremarkable. Genetic analysis, including whole-exome sequencing, mitochondrial DNA testing, and chromosome karyotype analysis, revealed no abnormalities. During the interictal period, brain PET/CT revealed hypometabolism in the right temporal and parietal lobes. She experienced a prolonged episode lasting for 6 h after admission. The ictal EEG displayed almost continuous generalized SW activity at 2.5–3 Hz, which gradually improved following intravenous administration of diazepam.

She was treated with VPA and OXC at the age of 13 and remained seizure-free for 3 years. At 17, she experienced another episode of GTCS, after which LTG was added to her treatment regimen, resulting in seizure-free status for 2 years. At 19, she began experiencing ASE at a frequency of 1–2 times per year. In response, the dosage of VPA was increased, and she remained seizure-free for 5 years. However, during a reduction of her medication, she experienced a recurrence of ASE, which occurred 1–3 times per month. Her treatment was then adjusted to LEV, VPA, and LTG, resulting in a seizure-free period of 18 months.

### 3.2 Clinical description

The clinical information, ancillary tests, treatment and outcomes were summarized in Table 1 in detail.

**Table 1:** Clinical features of three patients with ASE.

	Patient 1	Patient 2	Patient 3
Age/y	38	36	27
Gender	F	F	F
Personal medical history	(−)	Iron deficiency anemia for 14 years; hysteromyoma for 3 years	(−)
Age of ASE onset/y	21	22	19
Seizure types (frequency)	ASE (once a month); GTCS	ASE (once a month)	ASE (once or twice a month); GTCS
Duration of ASE	Several hours-24 h	6–24 h	24–48 h
Interictal EEG	(−)	(−)	(−)
Pre-ictal EEG	Gradually prolonged and more frequent generalized spike–wave discharges	Gradually prolonged and more frequent generalized spike–wave discharges	Gradually prolonged and more frequent generalized spike–wave discharges
Ictal EEG	2.5–3 Hz, continuous generalized spike-waves	2.5–3 Hz almost continuous generalized spike-waves	2.5–3 Hz almost continuous generalized spike-waves or spike waves
Precipitating factors	Menstruation	Menstruation	Menstruation
Physical examination of ictal period	Dilated pupils; slow light reflection; active tendon reflexes; Babinski sign (+); ankle clonus (+)	Confusion; aphasia; dilated pupils; extinguished light reflex; Babinski sign (−)	Confusion; muscle strength IV of upper limbs and III of lower limbs; Babinski sign (−)
Interictal MoCA	28	29	25
Interictal MMSE	29	27	29
MRI	(−)	(−)	(−)
PET-MR/PET-CT	Hypometabolism in the right temporal、parietal and occipital lobe, and the right cerebellum	Hypometabolism in the left hippocampus, temporal, parietal and occipital lobe	Decreased metabolism in the right temporal and parietal lobe
SUVavg of thalamus	Right: 8.195; left: 8.270	Right: 10.447; left: 9.722	
Aera of thalamus	Right: 247.878; left: 264.596	Right: 314.936; left: 308.672	
Gene test	(−)	NA	(−)
Treatment	VPA; LTG	LEV	LEV; VPA; LTG
Follow-up; prognosis	1 y; seizure-free	3 m, seizure-free	1.5 y; seizure-free

M/F, male/female; NA, not available; GTCS, generalized tonic–clonic seizures; ASE, absence status epilepsy; VPA, valproate; LTG, lamotrigine; LEV, levetiracetam; EEG, electroencephalogram; SUV, standard uptake value; avg, average.

The onset of ASE in our three cases occurred at a mean age of 20.67 ± 1.53 years, and all of them were female. The frequency of ASE was approximately once or twice per month, with durations ranging from several hours to 2 days. Menstruation was noted as a possible precipitating factor. Symptoms during the ictal period included confusion, unresponsiveness, and incontinence. Patients 1 and 3 had a history of GTCS. Ictal EEGs of all patients revealed generalized SW activities at 2.5–3 Hz. In light of ethical considerations, we did not perform ictal PET scans on our three patients. Instead, interictal FDG-PET imaging was performed, which revealed relative hypometabolism in the temporal, parietal, and occipital cortices and cerebellum. Additionally, a comparative analysis between the three patients and a control group of 10 healthy individuals demonstrated a significant reduction in the thalamic area and metabolic values. The control group exhibited a left thalamic area of 408.581mm^2^, a right thalamic area of 386.124 mm^2^, a left standard uptake value (SUVavg) of 13.235, and a right SUVavg of 13.401. The thalamic area and SUVavg of the three patients were shown in Table 1.

### 3.3 Literature review

We reviewed the clinical information of 17 previously reported cases from 7 studies (2, 3, 7, 8, 13–16). Together with our three cases, 20 patients with ASE were identified. The main data of all patients were shown in Supplementary Table 1.

The average age of ASE onset was 28.1 ± 15.07 years, with 55% of cases being female. The episodic frequency was relatively low, ranging from once per year to once per month, and the duration of each episode varied from 30 min to 3 weeks. The episodes of five patients could be precipitated by menstruation, and one was triggered by sleep deprivation and emotional disturbance. Ictal EEG of all patients revealed 2–4 Hz generalized SW discharge, and brain MRI was unremarkable.

Bilo et al. (7) initially described the findings of ictal ^18^F-FDG-PET, which revealed relative hypermetabolism in the bilateral thalamus and cerebellar vermis, along with hypometabolism in the bilateral frontal and parietal cortices and cerebellar hemispheres. In contrast, no abnormalities were detected in the interictal ^18^F-FDG-PET scan. Shimogori et al. (8) similarly described a female patient whose ictal ^18^F-FDG-PET showed hypermetabolism in the thalamus and cerebellar vermis, bilaterally and symmetrically distributed, as well as hypometabolism in the bilateral frontal and parietal cortices and the right temporal cortex.

## 4 Discussion

Due to the low incidence and insufficient knowledge of the disease, ASE is rarely reported both domestically and internationally. In this study, we considered ASE as a specific syndrome of GGEs, potentially influenced by multiple genes, despite the absence of abnormalities in gene testing among our patients. The onset of ASE in our patients typically occurred between adolescence and early to mid-adulthood, with recurrent typical AS being the predominant seizure type. In addition to typical AS, patients 1 and 3 also presented with GTCS, which started with their first seizure. These patients lacked a positive family history. Photoparoxysmal responses and activation in response to hyperventilation were infrequently observed. Ictal EEG detected 2.5–3 Hz generalized SW discharges, whereas the interictal EEG was normal. Impaired cognition and positive physical examination results were noted during the ictal periods; however, no significant cognitive decline was observed during the interictal periods. Notably, our patients experienced episodes closely correlated with menstruation, potentially acting as a precipitating factor. Most patients responded well to VPA and LTG, whereas OXC was found to increase the seizure frequency.

Among our three patients, the PET imaging of patients 1 and 2 demonstrated hypometabolism in the temporal, parietal, and occipital lobes, and patient 3 showed decreased metabolism in the temporal and parietal lobes. The underlying mechanism of this abnormal metabolism remains undetermined.

Gotman et al. (17) studied the metabolic pattern in patients with generalized 2–3 Hz SW discharge. Their findings revealed hypermetabolism (activation) in the thalamus and midfrontal regions, alongside symmetrical hypometabolism (deactivation) in the parietal and frontal regions. These results are consistent with studies by Bilo et al. (7) and Shimogori et al. (8), who also noted similarities between this metabolic pattern and the pattern observed when the default state of brain is suspended. When individuals are exposed to external stimuli or focus on tasks, the default state is disturbed, leading to cortical deactivation involving the frontoparietal cortical regions, including the posterior cingulate gyrus (18). Furthermore, the thalamus is crucial in the generation of generalized SW discharge (17), with thalamocortical networks playing a significant role in the pathophysiology of absence seizures (19). Consequently, during ictal periods in patients with ASE, this suspension of the default state of brain by generalized 2–4 Hz SW discharges, combined with decreased perception of sensory inputs and reduced responsiveness due to thalamic activation, may explain altered consciousness (17).

Unlike the normal interictal metabolism described by Bilo et al. (7), our patients exhibited hypometabolism in the posterior regions, including the temporal, parietal and occipital lobes. One possible explanation is that the PET/MRI examinations were performed close to their episodes. Furthermore, we observed that the hypometabolic regions align with the neural correlates of consciousness (NCC). The anatomical NCC was discussed by Koch et al. (20), who reported that the posterior cortices, rather than the broad frontoparietal network, are considered a more accepted candidate zone. Research has shown that patients in a minimally conscious state (MCS) exhibit notably reduced metabolism compared with healthy controls (21). Some studies have reported MCS*, which refers to patients who lack signs of consciousness, but display residual brain activity as evidenced by neuroimaging or neurophysiological data (22). Thibaut et al. (22) investigated the metabolic differences among patients in a vegetative state (VS), MCS*, and MCS. The results revealed that MCS* patients presented lower metabolism than MCS patients did, particularly in the posterior regions. The clinical manifestations of our three patients are similar to those of patients in the MCS*, characterized by a lack of response to commands and a decreased level of consciousness. Therefore, we concluded that the involvement of posterior regions may contribute to their clinical manifestations. The definite mechanism of ASE is unclear, and our findings offer insight into a potential mechanism of ASE.

We speculate that the thalamic area and SUVavg of patients are lower than those of healthy individuals for the following reasons. First, the connections between the thalamus and the cerebral cortex ensure the maintenance of consciousness, with disruptions in these connections potentially resulting in VS or MCS (23). Therefore, the decreased metabolic activity of the thalamus may lead to the interruption of consciousness. Moreover, Lutkenhoff et al. (24) found a correlation between thalamic atrophy and a decline in motor and communication functions, indicating that the unresponsiveness and inability to speak observed in our three patients may be attributed to a reduced thalamic area.

There are several limitations of our study. First, some patients with generalized activity on scalp EEG actually have a deep seizure focus when intracranial electrodes are placed. The lack of intracranial electrodes placement to preclude a deep seizure focus may be a potential limitation of this case report. However, the diagnosis of these three patients is based on widely accepted standardized definitions. And these three patients also became seizure-free for several months to 2 years by treatment with broad spectrum of anti-seizure medications. Therefore, it is unethical to place intracranial electrodes on these patients. Second, our patients only underwent interictal PET examination; thus, essential ictal PET results were lacking. Besides, the current studies also lack simultaneous EEG and PET assessments during the ictal period. Third, the reported cases are rare, and a larger sample size is still needed to further explore the underlying mechanism.

## 5 Conclusion

ASE is a rare condition and poses challenges in making a definite diagnosis. It is classified as a unique form of GGE, and an accurate diagnosis typically involves a comprehensive analysis of clinical manifestations, EEG findings, and even interictal PET scans. The onset of ASE occurs between adolescence and early to mid-adulthood, with recurrent typical AS being the predominant seizure type. Ictal EEG shows 2.5–3 Hz generalized SW discharges, whereas interictal EEG is normal. Recurrent seizures have no significant impact on the patients’ cognition. Menstruation may be a precipitating factor for ASE. VPA and LTG are considered the first choices for treatment. Interictal FDG-PET can reveal relative hypometabolism in the posterior cortices and a smaller thalamic area and metabolic value, which may be related to the important role of posterior regions in sustaining wakefulness. Additionally, the reduced thalamic area may lead to confusion and unresponsiveness in patients.

## Data Availability

The raw data supporting the conclusions of this article will be made available by the authors, without undue reservation.
