# Supplementary material for: The clinical features and 18F-FDG-PET analysis of absence status epilepsy
Source: Front Neurol. 2025 May 2;16:1521842. doi: 10.3389/fneur.2025.1521842 (PMC12081254; doi:10.3389/fneur.2025.1521842)
Supplement: Supplementary file 1 [file Table_1.docx]

| **Table 1. The clinical characteristics of 20 patients with absence status epilepsy.** | | | | | | | | | | | | |
| --- | --- | --- | --- | --- | --- | --- | --- | --- | --- | --- | --- | --- |
| Author, year | gender, age | personal medical history | age of ASE onset(y) | ASE frequency | duration of ASE | other types of seizure | precipitating factors | ictal EEG | MRI | PET-CT/PET-MR | treatment | follow-up; prognosis |
| Nightingale and Welch, 1982 | F,66 | (-) | 56 |  |  | GTCS |  |  |  | NA | PRM, PHT | persistence of seizures |
| Zambrellietal. 2006 | M, 83 | (-) | ＞20 | 2 | 48-72h | GTCS |  | sharp waves followed by a slow wave or by 3Hz SWD | NA | NA | LGT, VPA | seizure-free |
| Genton et al. 2008 | F,39 | (-) | 14 | 1/m-1/y | 4-32h | GTCS | associated with menstruation | 2-4Hz GSPWD |  | NA | VPA | 7 years; seizure-free |
|  | F,53 | (-) | 34 | 1/y | 10-48h | TAs |  | 3Hz GSPWD |  | NA | VPA, LTG | 2 years; seizure-free |
|  | F,41 | (-) | 16 | 2-3/y | 24-48h | GTCS |  | 2-4Hz GSPWD | (-) | NA | VPA, ETS, CLN | sporadic AS |
|  | M,53 | psoriasis | 35 | 2/m | 0.5-48h | GTCS |  | 3Hz GSPWD | (-) | NA | VAP, ETS | 11 years; seizure-free |
|  | M,44 | essential hypertension | 15 | 2/w | 0.5-3h | GTCS, eyelid myoclonia |  | 2-4Hz GSPWD, generalized SW and PSW discharges induced by hyperventilation | (-) | NA | VPA | 1 years; seizure-free |
|  | M,48 | (-) | 26 | several/y | several h | GTCS |  | 2-4Hz GSPWD, generalized SW and PSW discharges induced by hyperventilation | (-) | NA | VPA | 11 years; seizure-free |
|  | F,39 | (-) | 26 | 1/m | 3-24h | GTCS |  | 2-4Hz GSPWD, generalized SW and PSW discharges induced by hyperventilation | (-) | NA | VPA, PB, LEV | sporadic AS (stopped by CLB 40mg) |
|  | F,70 | osteoporosis dyslipidemia | 65 | 4 | several h | GTCS, negative myoclonus |  | 2-4Hz GSPWD | (-) | NA | VPA | 10 years; seizure-free |
|  | M,29 | late puberty | 16 | several/y | several h | GTCS, TAs, eyelid myoclonia, negative myoclonus |  | 2-4Hz GSPWD, generalized SW and PSW discharges induced by hyperventilation | (-) | NA | VPA, LTG | AS in the last 6 years |
|  | M,77 | (-) | 36 | 5 | 24h-2-3w | TAs |  | 2-4Hz GSPWD |  | NA | TPM, LEV | 6 months; seizure-free |
|  | M,62 | (-) | 36 | 1/m-1/y | 24-72h | GTCS, eyelid myoclonia with lip protrusion |  | 2-4Hz GSPWD | (-) | NA | LTG, ETS | 3 years; seizure-free |
| Bilo et al. 2010 | M,56 | (-) | 14 | 1/m | 36-48h | GTCS |  | 2-3Hz GSPWD | (-) | ictal: hypermetabolism in thalamus bilaterally and the cerebellar vermis, hypometabolism in bilateral frontal cortex, parietal and posterior cingulate cortices and in cerebellar hemispheres; interictal: normal | LTG, VPA | seizure-free |
| Pro et al. 2011 | F,72 | epilepsy family history | 54 | 2-3/y | several h | (-) |  | 3-4Hz GSPWD | slight subcortical brain atrophy with very mild brain ventricular dilation | NA | LTG | seizure-free |
| Lyer et al. 2014 | M,49 | (-) | 20 |  | several h | GTCS; | precipitated by sleep deprivation and emotional disturbance | 3Hz SW | (-) | NA | VPA | 6m, seizure-free |
| Shimogori et al. 2017 | F,17 | febrile seizure at age of 3 years(impairment of consciousness) | 17 |  | several h | (-) | during the perimenstruation period | continuous generalised 3-4Hz rhythmic delta waves | (-) | ictal: symmetrical hypermetabolism in the bilateral thalamus and cerebellum, hypometabolism in the frontal, parietal and posterior cingulate cortices and in the right temporal cortex | LTG, CZP |  |
| patient 1 | F,38 | (-) | 21 | 1/m | several h | GTCS | menstruation | 2-4Hz GSPWD | (-) | hypometabolism in the right temporal、parietal and occipital lobe, and the right cerebellum | VPA, LTG | 1y, seizure-free |
| patient 2 | F,36 | (-) | 22 | 1/m | 6-24 h | (-) | menstruation | 2-4Hz GSPWD | (-) | hypometabolism in the left hippocampus, temporal, parietal and occipital lobe | LEV | 3m, seizure-free |
| patient 3 | F,27 | (-) | 19 | 1-2/m | 24-48 h | GTCS | menstruation | 2-4Hz GSPWD | (-) | decreased metabolism in the right temporal and parietal lobe | LEV, VPA, LTG | 1.5y, seizure-free |
| Note: M/F, male/female; NA, not available; m, month; y, year; h, hour; w, week; EEG, electroencephalogram; GTCS, generalized tonic-clonic seizures; ASE, absence status epilepsy; TAs: typical absences; GSPWD, generalized spike/poly spike and wave discharges; VPA, valproate; LTG, lamotrigine; LEV, levetiracetam; PRM, primidone; PHT, phenytoin; ETS, ethosuximide; CLN, clonazepam; PB, phenobarbital; TPM, topiramate; CZP, clonazepam; | | | | | | | | | | | | |
